# SMARCB1-deficient sinonasal adenocarcinoma: a rare variant of SWI/SNF-deficient malignancy often misclassified as high-grade non-intestinal-type sinonasal adenocarcinoma or myoepithelial carcinoma

**DOI:** 10.1007/s00428-023-03650-2

**Published:** 2023-12-12

**Authors:** Alena Skálová, Touraj Taheri, Martina Bradová, Tomáš Vaněček, Alessandro Franchi, David Slouka, Tomáš Kostlivý, Gisele de Rezende, Jaroslav Michálek, Natálie Klubíčková, Nicola Ptáková, Antónia Nemcová, Michal Michal, Abbas Agaimy, Ilmo Leivo

**Affiliations:** 1 Department of Pathology, Faculty of Medicine in Pilsen, Charles University, E. Benese 13, 305 99 Pilsen, Czech Republic; 2 Bioptic Laboratory, Ltd., Pilsen, Czech Republic; 3 Department of Anatomical Pathology, Queensland Health, Royal Brisbane and Women Hospital, University of Queensland, Brisbane, Australia; 4 Molecular and Genetic Laboratory, Bioptic Laboratory, Ltd, Pilsen, Czech Republic; 5 Department of Translational Research, School of Medicine, University of Pisa, Pisa, Italy; 6 Department of Otorhinolaryngology, University Hospital in Pilsen, Faculty of Medicine in Pilsen, Charles University, Pilsen, Czech Republic; 7 Department of Anatomic Histopathology and Cytogenetics, Department of Laboratory Medicine, Niguarda Cancer Center, Milan, Italy; 8 Department of Clinical and Molecular Pathology, University Hospital and Medical Faculty of Palacky University, Olomouc, Czech Republic; 9 Pathological Laboratories, Medicyt, Ltd., Košice, Slovak Republic; 10 Institute of Pathology, University Hospital Erlangen, Friedrich‐Alexander University Erlangen‐Nürnberg (FAU), Erlangen, Germany; 11 Institute of Biomedicine, Pathology, University of Turku and Department of Pathology, Turku University Hospital, Turku, Finland

**Keywords:** SWI/SNF complex, SMARCB1-deficient adenocarcinoma, Sinonasal, Head and neck, Rhabdoid, Yolk sac-like, Next-generation sequencing

## Abstract

SMARCB1-deficient sinonasal adenocarcinoma is a rare variant of SWI/SNF-deficient malignancies with SMARCB1 loss and adenocarcinoma features. More than 200 high-grade epithelial sinonasal malignancies were retrieved. A total of 14 cases exhibited complete SMARCB1 (INI1) loss and glandular differentiation. SMARCA2 and SMARCA4 were normal, except for one case with a loss of SMARCA2. Next-generation sequencing (NGS) and/or fluorescence in situ hybridization (FISH) revealed an alteration in the *SMARCB1* gene in 9/13 cases, while 2/13 were negative. Two tumors harbored *SMARCB1* mutations in c.157C > T p.(Arg53Ter) and c.842G > A p.(Trp281Ter). One harbored *ARID1B* mutations in c.1469G > A p.(Trp490Ter) and *MGA* c.3724C > T p.(Arg1242Ter). Seven tumors had a *SMARCB1* deletion. One carried an *ESR1* mutation in *c.644-2A* > *T*, and another carried a *POLE* mutation in c.352_374del p.(Ser118GlyfsTer78). One case had a *PAX3* mutation in c.44del p.(Gly15AlafsTer95). Histomorphology of SMARCB1-deficient adenocarcinoma was oncocytoid/rhabdoid and glandular, solid, or trabecular in 9/14 cases. Two had basaloid/blue cytoplasm and one showed focal signet ring cells. Yolk sac tumor-like differentiation with Schiller-Duval-like bodies was seen in 6/14 cases, with 2 cases showing exclusively reticular-microcystic yolk sac pattern. Follow-up of a maximum of 26 months (median 10 months) was available for 8/14 patients. Distant metastasis to the lung, liver, mediastinum, bone, and/or retroperitoneum was seen in 4/8 cases. Locoregional failure was seen in 75% of patients, with 6/8 local recurrences and 3 cervical lymph node metastases. At the last follow-up, 5 of 8 (62%) patients had died of their disease 2 to 20 months after diagnosis (median 8.2 months), and 3 were alive with the disease. The original diagnosis was usually high-grade non-intestinal-type adenocarcinoma or high-grade myoepithelial carcinoma. A correct diagnosis of these aggressive tumors could lead to improved targeted therapies with potentially better overall disease-specific survival.

## Introduction

Poorly differentiated or undifferentiated sinonasal carcinomas represent a challenging area in head and neck pathology. Over the recent years, advances in molecular techniques have led to significant progress in understanding the molecular underpinnings of sinonasal malignancies, with significant developments in the histological and pathogenetic classification of entities included in the historical spectrum of “sinonasal undifferentiated carcinoma (SNUC)” and poorly differentiated unclassified carcinoma [1]. SNUC, a diagnosis of exclusion, should be made only in cases of relatively monomorphic, sometimes basaloid-looking, high-grade/poorly differentiated tumor cells with evidence of epithelial origin and the absence of features pointing toward other entities. Recently, up to 80% of SNUC cases were reported to harbor hotspot mutations in the *IDH2* gene [2, 3], while mutations in the *IDH1* gene have been reported only rarely [3]. Another novel entity with a very poor prognosis is NUT carcinoma, composed of undifferentiated primitive cells with irregular overlapping nuclei with prominent nucleoli and defined by pathogenic fusions of the *NUTM1* gene, most commonly *NUTM1::BRD4* [4].

Recent molecular-genetic findings have aided in subclassifying primary sinonasal carcinomas, prompting the inclusion of several new entities in the 5th edition of the *WHO Classification of Head and Neck Tumors* [5]. A major change has been the recognition of subtypes of carcinomas defined by genetic defects leading to the inactivation of different protein subunits in the switch/sucrose nonfermentable (SWI/SNF) chromatin remodeling complex [6]. This led to the identification of 4 sinonasal entities driven by SWI/SNF deficiency complexes: SMARCB1 (INI1)-deficient sinonasal carcinoma (lacking gland formation and frequently displaying a non-descript basaloid and less frequently eosinophilic/oncocytoid morphology), SMARCB1-deficient sinonasal adenocarcinoma with unequivocal glands or yolk sac-like pattern, SMARCA4-deficient carcinoma lacking glandular or squamous immunophenotypes, and SMARCA4-deficient subset (~ 80%) of sinonasal teratocarcinosarcoma [6]. The most common of these four, the SMARCB1 (INI-1)-deficient sinonasal carcinoma, is an aggressive tumor that was first reported in 2014 independently by two research groups [7, 8]. It is a rare form of cancer arising in the nasal cavity and paranasal sinuses, and it is characterized by the loss or inactivation of the SWI/SNF-complex genes that play crucial roles in the regulation of cell growth and division [9].

Primary sinonasal adenocarcinomas (SNAC) are rare tumors encompassing a wide morphological spectrum. They are divided into three groups: intestinal-type sinonasal adenocarcinoma (ITAC), non-intestinal-type sinonasal adenocarcinoma (non-ITAC), and adenocarcinomas of salivary gland subtypes [10]. The classification of SNAC has developed in the last two decades [11]. High-grade non-intestinal SNACs are particularly heterogeneous, with highly variable morphology [12] including several molecularly defined new entities [13, 14].

SMARCB1-deficient sinonasal adenocarcinoma is a rare SWI/SNF-deficient malignancy defined by the presence of unequivocal glandular differentiation and/or by the presence of other features of adenocarcinoma [15]. Glandular differentiation has been very rarely reported in SMARCB1 (INI-1)-deficient sinonasal carcinomas, although it is possible that this feature has been underrecognized [16–19]. The tumors are usually composed of oncocytoid/plasmacytoid tumor cells in rounded nests, trabeculae, reticular, microcystic, and glandular patterns and could be misdiagnosed as high-grade non-intestinal adenocarcinoma, myoepithelial carcinoma, or even metastatic yolk sac tumor [20].

The use of next-generation sequencing platforms in clinical practice has allowed for further subclassification of tumors previously grouped together. The discovery of a subset of tumors with several unique molecular features has opened up the door to potential therapeutic targets [21]. These targetable subsets include not only NUT carcinoma [22] and SNUC with a novel isocitrate dehydrogenase (IDH) mutation [23] but also SWI/SNF-deficient sinonasal malignancies [24]. We aim to summarize our experience with a subset of SWI/SNF-deficient sinonasal adenocarcinomas diagnosed in three major consult centers in head and neck pathology (AS, AA, and AF).

## Materials and methods

### Case selection

In their consultation practice, two of the authors (AA and AS) encountered cases of sinonasal undifferentiated malignancies reminiscent of salivary myoepithelial carcinoma or high-grade sinonasal non-ITAC that showed a complete loss of *SMARCB1* immunoexpression. Indeed, some of these cases were submitted for consultation with a diagnosis of sinonasal myoepithelial carcinoma or high-grade sinonasal non-ITAC, and a diagnosis of SMARCB1-deficient adenocarcinoma was not raised by the primary pathologist. These consult cases prompted us to perform a computer search of our routine and consult files for high-grade sinonasal non-ITAC carcinomas and SNUCs. Seventy-five and one hundred thirty high-grade malignant tumors of sinonasal localization with epithelial features and available tissue material were retrieved from the authors’ files, respectively (AS and AA). All cases were evaluated morphologically and examined immunohistochemically (IHC) by cytokeratin cocktail AE1-AE3 antibodies and antibodies to CK7, CK5/6, p63/p40, SOX10, S100, SALL4, glypican-3, and SWI/SNF proteins (Table 1). Next-generation sequencing (NGS) using the Illumina Oncology TS500 and TST170 DNA panels was performed in 44 cases, including 9 cases that demonstrated loss of SWI/SNF proteins by immunohistochemistry (AS) and 4 cases (AA). The latter cases were subjected to selected immunohistochemical and molecular studies (Table 2).

**Table 1:** Antibodies used for immunohistochemical study

Antibody specificity	Clone	Dilution	Antigen retrieval/time	Source
AE1/3	AE1/AE3 + PCK26	RTU	CC1/20 min	Ventana
CK7	OV-TL 12/30	RTU	EnVision High pH/30 min	Dako
CK5/6	D5/16B4	1:50	EnVision High pH/30 min	Dako
p63	DAK-p63	RTU	EnVision Low pH/30 min	Dako
p40	BC28	RTU	CC1/52 min	Biocare Medical
SOX 10	SP267	RTU	CC1/64 min	Cell Marque
S100	Polyclonal	RTU	EnVision High pH/30 min	Dako
CDX2	EPR2764Y	RTU	CC1/64 min	Cell Marque
CK20	Ks20.8	1:100	CC1/36 min	DakoCytomation
MIB1	30–9	RTU	CC1/64 min	Ventana
SALL4	6E3	1:800	CC1/64 min	Sigma-Aldrich
Glypican-3	GC33	RTU	CC1/52 min	Ventana
SMARCA2	Polyclonal	1:200	CC2/56 min	Atlas Antibodies AB
SMARCA4	EPNCIR111A	1:1000	CC1/52 min	Abcam
SMARCB1	MRQ-27	RTU	CC1/52 min	Ventana

*RTU*, ready to use; *CC1*, EDTA buffer pH 8.6 at 95 °C; *EnVision High*, pH 9.0 at 97 °C; *EnVision Low*, pH 6.0 at 97 °C; *min*, minutes

**Table 2:** Clinicopathological and molecular features of SMARCB1-deficient sinonasal adenocarcinomas, Pilsen (case 1–9); Erlangen (cases 10–14)

No	Age/sex	Site	Original diagnosis	Glandular pattern (% of glandular component)	Non-glandular pattern; predominant histology	IHC +	IHC −	Molecular analysis
1	66/M	Nasal cavity	Squamous cell carcinoma; HGT of acinic cell carcinoma	Oncocytoid, plasmacytoid, clear cells, abortive glandular (20%), tubular (10%)	Eosinophilic, oncocytoid; solid alveolar	AE1/AE3, CK7, EMA	CK20, CDX2, SOX10, S100, GATA3, NUT, p16 Glypican-3; SALL4	*TSO500: SMARCB1* c.157C > T p.(Arg53Ter), *ARID1B* c.1469G > A p.(Trp490Ter), *MGA* c.3724C > T p.(Arg1242Ter) FISH *SMARCB1:* not done
2	62/M	Ethmoid sinus	SNUC; HG non-ITAC	Abortive glandular (70%)	Rhabdoid, oncocytoid; solid alveolar	AE1/AE3, CK7, EMA	CK20, CDX2, SOX10, S100, GATA3, NUT, AR, desmin, MyoD1 Glypican-3; SALL4	TSO500: *SMARCB1* c.842G > A, p.(Trp281Ter) FISH *SMARCB1:* not done
3	39/M	Nasal cavity	SNUC; HG myoepithelial ca	Dyscohesive pattern, signet ring cell, large eosinophilic oncocytoid (60%)	Rhabdoid, solid alveolar	AE1/AE3, CK7, CK19, EMA, wk. p63	CK20, CDX2, AR, SOX10, S100, synaptophysin Glypican-3; SALL4	Not analyzable*
4	55/M	Nasal cavity	HG myoepithelial ca	Oncocytoid glandular (10%)	Eosinophilic, solid	AE1/AE3, OSCAR, CK7, wk. synaptophysin Glypican-3	CK14, CK20, CDX2, S100; SALL4 Chromogranin, CK5/6, p16, myogenin, MyoD1, SOX10, CD56, CD99	Not analyzable*
5	66/M	Nasal cavity	SMARCB1-deficient adenocarcinoma	Yolk sac-like structures with Schiller-Duval-like bodies (90%)	Basaloid to eosinophilic, solid	Glypican-3, wk. CDX2, AE1-AE3, OSCAR, CAM.5.2	SALL4, NUT, p16	TSO500: *SMARCB1* heterozygous deletion, *ESR1* c.644-2A > T FISH *SMARCB1*: heterozygous deletion**
6	36/M	Nasal cavity	HG ITAC	Yolk sac-like structures with Schiller-Duval-like bodies (85%), abortive glandular (5%)	Eosinophilic, solid	Glypican-3; SALL-4; AE1-AE3; wk. CDX2	CK7; CK20	TSO500: *SMARCB1* heterozygous deletion FISH *SMARCB1*: homozygous deletion***
7	Xx/M	Nasal cavity	Unclassified carcinoma; HG myoepithelial ca	Oncocytoid; glandular (10%), abortive glandular (60%)	Eosinophilic, rhabdoid, oncocytoid; solid trabecular, clear cell	AE1/AE3, OSCAR, CK7, CK19, p63	Glypican-3; SALL4; p40, p16, SOX10	TSO500: negative FISH SMARCB1: negative
8	46/M	Maxillary sinus	SMARCB1-deficient adenocarcinoma	Yolk sac-like structures with Schiller-Duval-like bodies (70%), glandular (10%)	Basaloid, rhabdoid, solid alveolar	P63, OSCAR, CK5/6, CK7, glypican-3	S100, desmin, synaptophysin, NUT, SOX10, p16, SALL-4	VST: *SMARCB1* heterozygous deletion FISH SMARCB1: not analyzable
9	52/M	Nasal cavity	SNUC; HG myoepithelial ca	Cribriform with minor lumens (20%)	Oncocytoid, eosinophilic, solid, clear cell	CK7, wk. p63, AE1-AE3,	S100, CK14, SOX10, p16, NUT, glypican-3; SALL4; INSM1, CD56	TSO500: not analyzable FISH *SMARCB1*: heterozygous deletion
10	62/M	Sinonasal site NOS	Carcinoma with INi1 loss	Abortive glandular with mucin	Eosinophilic, oncocytoid; solid areas	AE1/AE3, CK7, wk. synaptophysin	CK5/6, SALL4, S100, SOX10, AFP HepPar1, glypican-3, CK20, CDX2, p40	TS500: PAX3 c.44del p.(Gly15AlafsTer95) FISH *SMARCB1:* not done
11	76/F	Paranasal sinus right	Unclassified carcinoma	Pure reticular-microcystic yolk sac pattern		AE1/AE3, SALL4 & glypican-3 diffuse, focal p63, CK7, EMA & AFP	CD34, CD117, ERG, ALK, NUT, SOX10	TS500: SMARCB1 homozygous deletion, POLE c.352_374del p.(Ser118GlyfsTer78) FISH *SMARCB1:* not done
12	59/M	Sinuses left	SNUC or NUT carcinoma	Oncocytoid abortive glandular	Eosinophilic, trabecular	p63, AE1/AE3, focal CK5/6, wk. synaptophysin	CD34, CD117, CK7, CDX2, CD56, NUT, SOX10	TS500: SMARCB1 homozygous deletion FISH *SMARCB1*: homozygous deletion
13	NA	Sinonasal site NOS	HG non-ITAC	Abortive glandular	Solid, large cells	AE1/AE3 diffuse, CK7 variable, p63 focal	AR, S100, SOX10	TS500: not done FISH *SMARCB1:* not done
14	55/F	Nasal cavity + sinuses	Unclassified carcinoma	Reticular-myxoid, cribriform		AE1/AE3, SALL4	AFP	TS500: SMARCB1 homozygous deletion FISH *SMARCB1*: homozygous deletion

*Ca*, carcinoma; *F*, female; *FISH*, fluorescence in situ hybridization; *HG*, high grade; *HGT*, high-grade transformation; *ITAC*, intestinal type adenocarcinoma; *M*, male; *meta*, metastasis; *mo*, month; *NA*, not analyzable; *ND*, not done; *NED*, no evidence of disease; *NOS*, nor otherwise specified; *SNUC*, sinonasal undifferentiated carcinoma; *wk*, weak and/or focal staining; *TSO500*, TruSight Oncology 500; *VST*, VariantPlex Solid Tumor Panel; FISH—*SMARCB1* enumeration

^*^Not analyzable by all mentioned methods. **Atypical FISH pattern—susp. partial deletion of *SMARCB1* gene. ***Mix of tumor and non-tumor tissue (FISH counted in tumor tissue)

### Histological and immunohistochemical studies

For conventional microscopy, the excised tissues were fixed in formalin, processed routinely, embedded in paraffin (FFPE), cut, and stained with hematoxylin and eosin.

For immunohistochemistry, 4-μm-thick sections were cut from paraffin blocks and mounted on positively charged slides (TOMO, Matsunami Glass IND, Osaka, Japan). Sections were processed on a BenchMark ULTRA (Ventana Medical Systems, Tucson, AZ), deparaffinized, and subjected to heat-induced epitope retrieval by immersion in a CC1 solution (pH 8.6) at 95 °C. All primary antibodies used in this study are summarized in Table 1.

Visualization was performed using the ultraView Universal DAB Detection Kit (Roche, Tucson, AZ) and the ultraView Universal Alkaline Phosphatase Red Detection Kit (Roche, Tucson, AZ). The slides were counterstained with Mayer’s hematoxylin. Appropriate positive and negative controls were employed.

### Molecular studies

#### TruSight Oncology 500 Kit (TS500)

Mutation analysis and fusion transcript detection were performed using the TruSight Oncology 500 Kit (Illumina, San Diego, CA). RNA was extracted using the Maxwell RSC DNA FFPE Kit and the Maxwell RSC Instrument (Promega, Madison, WI) according to the manufacturer’s instructions and quantified using the Qubit HS RNA Assay Kit (Thermo Fisher Scientific, Waltham, MA). DNA was extracted using the QIAsymphony DSP DNA mini (Qiagen, Hilden, Germany) and quantified using the Qubit BR DNA Assay Kit (Thermo Fisher Scientific, Waltham, MA). The quality of DNA was assessed using the FFPE QC kit (Illumina) and the quality of RNA using the Agilent RNA ScreenTape Assay (Agilent, Santa Clara, CA). DNA samples with Cq < 5 and RNA samples with DV200 ≥ 20 were used for further analysis. After DNA enzymatic fragmentation with the KAPAFrag Kit (KAPA Biosystems, Wilmington, MA), DNA and RNA libraries were prepared with the TruSight Oncology 500 Kit (Illumina) according to the manufacturer’s protocol. Sequencing was performed on the NovaSeq 6000 sequencer (Illumina) following the manufacturer’s recommendations. Data analysis was performed using the TruSight Oncology 500 v2.2 Local App (Illumina). Variant annotation and filtering were performed using the Omnomics NGS analysis software (Euformatics, Espoo, Finland). A custom variant filter was set up including only non-synonymous variants with coding consequences, read depth greater than 50, and benign variants according to the ClinVar database [25] were also excluded. The remaining subset of variants was checked visually, and suspected artefactual variants were excluded.

#### VariantPlex Kit (VST)

DNA was extracted using the Qiasymphony DSP DNA mini (Qiagen, Hilden, Germany), and input DNA clean-up was performed using the Archer PreSeq™ DNA QC Assay Protocol. DNA was quantified using the Qubit HS DNA Assay Kit (Thermo Fisher Scientific, Waltham, MA, USA). The Archer VariantPlex Solid Tumor Kit was used (VST, ArcherDX Inc., Boulder, CO). Library preparation was performed following the Archer VariantPlex™ Protocol for Illumina (ArcherDx) and the product Insert VariantPlex™ Solid Tumor Panel. Final libraries were diluted 1:100,000 and quantified following the Library Quantification for Illumina (KAPA Biosystems, Wilmington, MA), normalized, and pooled. The libraries were sequenced on NovaSeq 6000 (Illumina) following the manufacturer’s recommendations. Variant annotation and filtering were performed using Archer Analysis software v7 (ArcherDx) with the parameters mentioned above.

#### Fluorescent in situ hybridization (FISH)

For the detection of *SMARCB1* deletion, ZytoLight® SPEC SMARCB1/22q12 Dual Color Probe was used (ZytoVision GmbH, Bremerhaven, Germany). The FISH procedure was performed as described elsewhere [26]. The heterozygous deletion was defined as one green signal (SMARCB1) compared to two orange control signals (KREMEN1), and the homozygous deletion as zero green signals (SMARCB1) compared to at least one orange control signal (KREMEN1).

## Results

### Demographic and clinical features

**Fig. 1:**
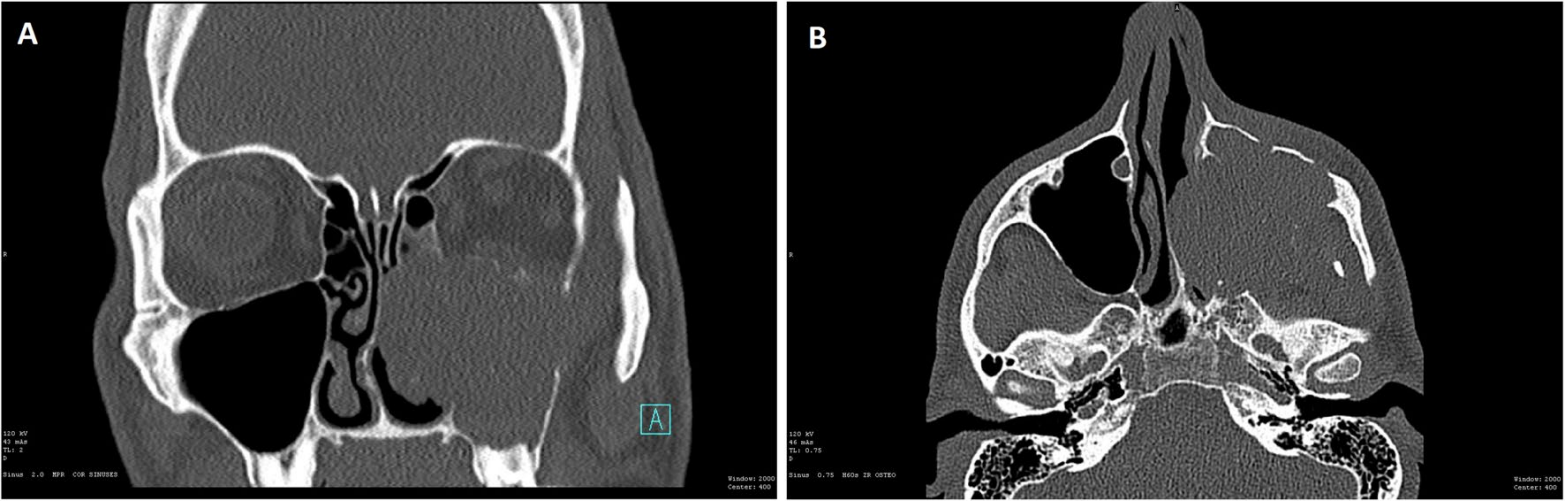
SMARCB1-deficient sinonasal adenocarcinoma T3N0M0: coronal noncontrast CT scan (**A**) and axial noncontrast CT scan (**B**)

A total of 14 cases demonstrated clear morphologic evidence of glandular differentiation and loss of SMARCB1 immunoexpression, and these were included in the study (Table 2). They occurred in 11 men and 2 women with ages ranging from 36 to 92 years (mean 59 years). Clinical data were not available for one patient. The tumors arose in the nasal cavity (7), maxillary sinus (1), ethmoid sinus (1), paranasal sinus NOS (4), and sinonasal tract NOS (1). Imaging revealed extensive involvement of the paranasal sinuses with or without the involvement of the nasal cavity and frequent involvement of the skull base (Fig. 1a, b).

Of eight patients with detailed tumor staging information, six (75%) presented with stage T4 disease with extensive involvement of the bony confinements of the sinonasal cavities and variable infiltration into periorbital or skull base tissues. Synchronous regional lymph node involvement and distant metastases were detected in one patient (Table 3).

**Table 3:** Clinical course and follow-up of SMARCB1-deficient sinonasal adenocarcinomas, Pilsen (cases 1–9); Erlangen (cases 10–14)

No	Age/sex	Initial stage	Treatment	Clinical course (months)	Outcome/follow-up (months)
1	66/M	pT4a, cN0, cM0	Radical surgery, CT	Recurrence (6) Cervical LN meta (6)	DOD (10)
2	62/M	pT4, cN0, cM0	No surgery, palliative RT, CT	Meta lungs (12)	DOD (20)
3	39/M	pT4, cN2, cM2	Surgery, RT, CT	Meta liver, LN, skeleton, retroperitoneum (2)	DOD (2)
4	55/M	Unknown	Unknown	Unknown	Unknown
5	66/M	pT4a, cN0, M0	Radical surgery, RT	Recurrent tumor (7)	AWD (7)
6	36/M	pT4a, cN0, M0	Surgery, RT, CT		AWD (6)
7	92/M	pT3, cN0, M0	Surgery, RT	Recurrent tumor (6)	DOD (6)
8	46/M	pT3, cN0, M0	Surgery, RT, proton	Recurrent tumor, meta lungs and mediastinum (24)	AWD (26)
9	52/M	cT4a, cNX, MX	Surgery, RT, CT	Recurrent tumor, meta cervical LN	DOD (3)
10	62/M	*	*	*	*
11	76/F	*	*	*	*
12	59/M	*	*	*	*
13	NA	*	*	*	*
14	55/F	*	*	*	*

*AWD*, alive with disease; *DOD*, died of disease; *CT*, chemotherapy; *F*, female; *LN*, lymph node; *M*, male; *meta*, metastasis; *NA*, not available; *NED*, no evidence of disease

^*^Clinical and follow-up data for cases 10–14 are not available

Treatment consisted of radical surgical resection combined with chemotherapy and/or radiation in eight patients. One patient received only supportive (palliative) care after a diagnostic biopsy. For the remaining six patients, detailed information regarding therapy was not available (Table 3).

Follow-up data were available for 8 patients, and the follow-up period ranged from shortly after diagnosis to 26 months (median, 10). Distant metastases were recorded in 4 of 8 cases (50%). The sites of distant metastases included the lungs (*n* = 2), the liver (*n* = 1), the mediastinum (*n* = 2), the bones (*n* = 1), and the retroperitoneum (*n* = 1). They occurred from presentation to 24 months after diagnosis (median, 10 months). Regional failure was seen in 75% of patients, with 6/8 local recurrences and 3 regional recurrences to cervical lymph nodes. At the last follow-up, 5 of 8 (62%) patients had died of their disease 2 to 20 months after diagnosis (median, 8.2 months), and 3 were alive with the disease (Table 3).

### Histopathological and immunohistochemical finding

**Fig. 2:**
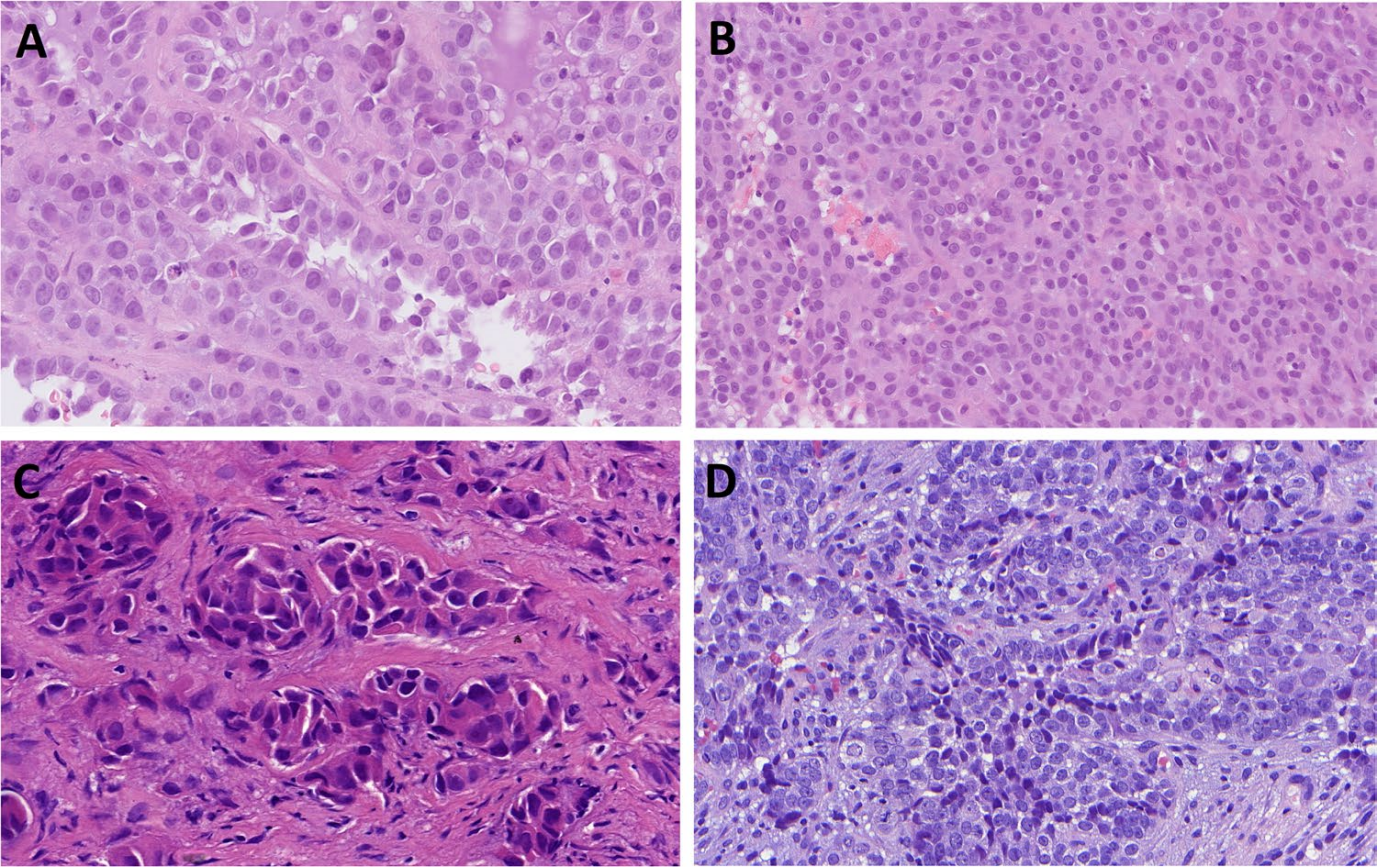
SMARCB1-deficient sinonasal adenocarcinoma is characterized by the presence of oncocytoid/plasmacytoid cell morphology, with variable but unequivocal gland formation (**A**–**C**) defined by open glandular structures (**A**), abortive microglandular differentiation (**B**), and small nests (**C**). Less common pattern is basaloid/blue phenotype with uniformly high-grade cytomorphology (**D**)

**Fig. 3:**
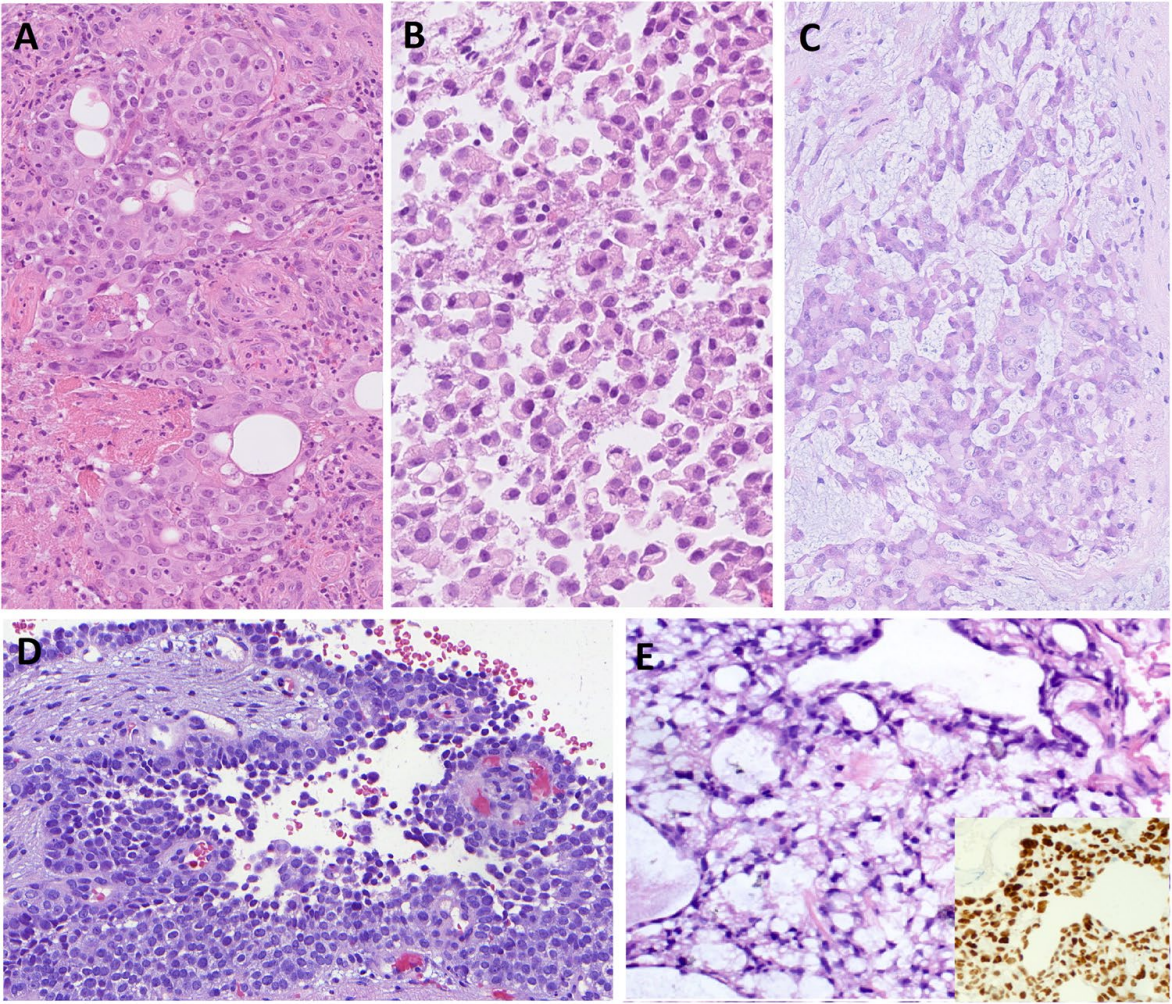
Cribriform structures may be present in solid areas (**A**), and focal signet-ring cell pattern was noted in one case (**B**). Fibrous stroma with intermingled glands with a sieve-like morphology or floating tumor cell strips embedded within mucoid stroma was present in two cases (**C**) along with structures resembling the Schiller-Duval bodies. (**D**) Two cases showing exclusively reticular-microcystic yolk sac pattern (**E**) positive for SALL4 (inset)

In most of the cases, tumor histomorphology was predominantly solid, with trabecular and alveolar growth patterns. The tumor cells were large with eosinophilic, oncocytoid, plasmacytoid, and/or rhabdoid appearance. In nine cases (9/14, 64%), the dominant cell morphology was oncocytoid/rhabdoid (Fig. 2a–c). However, in two cases a basaloid/blue cytoplasm was observed (Table 2 and Fig. 2d). All cases demonstrated varying proportions of glandular changes, including alveolar/acinar with abortive microglandular differentiation, trabecular, and solid/cribriform/insular patterns. A focal signet-ring cell pattern was noted in one case (Table 2 and Fig. 3a, b). Variable luminal and stromal mucin-like secretion was noted in some cases, one case with prominent myxoid stroma. Areas with yolk sac tumor-like differentiation with Schiller-Duval body-like structures were found in six cases (6/14; 43%) with two cases showing exclusively reticular-microcystic yolk sac pattern (Table 2 and Fig. 3c–e). Seven cases with high-grade malignant histomorphology and two cases with intermediate-grade malignant histomorphology were observed (Table 2). In all cases, the Ki-67 index was high (> 40%).

**Fig. 4:**
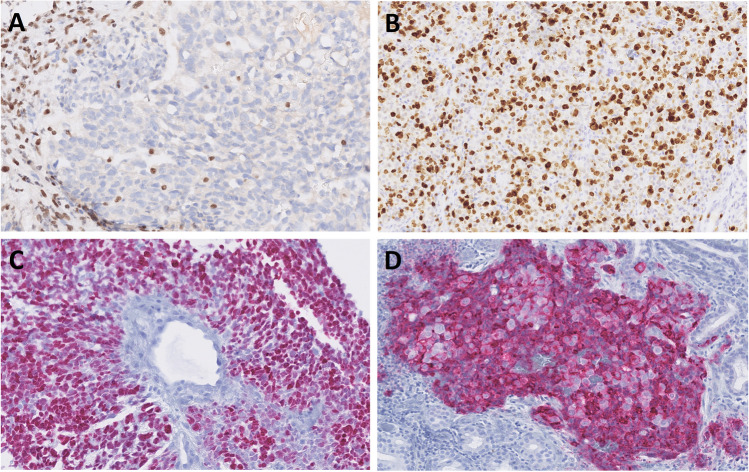
Representative example of complete loss of SMARCB1 immunohistochemistry in tumor cells with positive internal control in lymphocytes and fibroblasts (**A**). Proliferative activity was high with Ki-67 > 40% (**B**). Germ cell markers of yolk sac-like differentiation, SALL4 (**C**) and glypican-3 (**D**) highlighted 6 cases

In immunohistochemical stainings, all cases were completely negative for SMARCB1 (INI-1) proteins (Fig. 4a). SMARCA2 and SMARCA4 had normal patterns of expression (retained) except for case 14, which has shown SMARCA2 loss. In all cases, the neoplastic cells showed strong staining for cytokeratins AE1/AE3 and/or OSCAR (9/9), CK7 (9/9), and EMA (4/4), while S100, SOX10, CK20, CDX2, p63, p40, GATA3, NUT, and AR were mostly negative. Proliferative activity was high, with the Ki-67 index reaching 40–80% (mean 56%) (Fig. 4b). Focal p63 staining was seen in 5 cases, CDX2 staining in 2 cases, and weak focal synaptophysin in 3 cases. Immunohistochemical markers for yolk sac tumor (SALL4 or glypican-3) decorated 6 cases (Fig. 4c–d), corresponding to their yolk sac tumor-like histologies.

### Molecular findings

Next-generation sequencing (NGS) and/or fluorescence in situ hybridization (FISH) revealed alteration in the *SMARCB1* gene in nine cases (9/13, 69%), while two cases were negative and two cases were not analyzable. In one case, molecular analysis was not done. (Table 2). Among the positive cases, two had nonsense mutations truncating the *SMARCB1* gene, namely *SMARCB1* c.157C > T p.(Arg53Ter) and c.842G > A, p.(Trp281Ter). Seven cases had deletions in the *SMARCB1* gene detected by NGS or FISH. One case was negative for both SNV and CNV analysis by NGS and also negative for *SMARCB1* deletion by FISH. Another one was negative for SNV and CNV by NGS only. In addition, one of these tumors harbored mutations in genes *ARID1B* c.1469G > A p.(Trp490Ter) and *MGA* c.3724C > T p.(Arg1242Ter); another case had a splicing mutation in the *ESR1* gene c.644-2A > T and the other had a mutation in gene *POLE* c.352_374del p.(Ser118GlyfsTer78). In addition, in one case, there was a mutation in the gene *PAX3* c.44del p.(Gly15AlafsTer95). The complete results of molecular testing are illustrated in Table 2.

## Discussion

SWI/SNF (switching/sucrose non-fermentable) genes were first described in *Saccharomyces* yeast in 1984 as genes required to enable mating-type switching and sucrose metabolism [27]. In the process of evolution from yeast to mammals, the SWI/SNF complex (aka BAF complex) has evolved into a large ATP-dependent chromatin remodeling complex with many subunits encoded by a variety of genes with a markedly heterogeneous structure and function. Due to the tumor suppressor role of this complex, somatic mutations in SWI/SNF genes are involved in the tumorigenesis of multiple human neoplasms [28, 29]. Other tumor suppressor genes, such as *RB1*, *TP53*, *MYC*, and *BRCA1*, are known to interact with the SWI/SNF complex, and therefore mutations in these partner genes can affect its function and increase cell proliferation [28–31]. Up to 25% of human cancers carry mutations in at least one of nine SWI/SNF subunit genes including *SMARCA1* and *2*, *SMARCB1*, *ARID1A/B*, *PBRM1*, and *ARID2* [32].

SMARCB1 (SWI/SNF-related, matrix-associated, actin-dependent regulator of chromatin, subfamily b, member 1) is also known as INI1 (for integrase interactor 1) and SNF5, the latter of which was initially named based on a conserved region of this protein in the SWI/SNF complex in yeast [33]. *SMARCB1*, located on chromosome 22q11.2, functions as a tumor suppressor gene. Mutations in the *SMARCB1* gene were first described in rhabdoid tumors in 1998, evidencing for the first time the link between the SWI/SNF complex and human cancer [33]. *SMARCB1* mutations have been implicated in the pathogenesis of several malignancies, including atypical teratoid rhabdoid tumor, malignant rhabdoid tumor, epithelioid sarcoma, renal medullary carcinoma, myoepithelial carcinoma, epithelioid malignant peripheral nerve sheath tumor, and extraskeletal myxoid chondrosarcoma [8]. Although these diverse neoplasms exhibit many distinct clinicopathologic features, they all tend to share the presence of “rhabdoid” cells, defined as large cells with abundant eosinophilic cytoplasm and eccentrically placed nuclei with open chromatin and prominent nucleoli. A meta-analysis of 10,849 patients from 15 studies found that 5% of human cancers had alterations in SMARCB1 [34, 35, 36]. Such tumors mostly but not exclusively comprise rare, high-grade lethal cancers [32, 34].

Since the first description of malignant rhabdoid tumors in pediatric patients, various histological patterns in SWI/SNF-mutated tumors have been reported. Such tumors comprise, but are not limited to, rhabdoid eosinophilic cells, blue-basaloid cells, small cells, and clear cells, as well as glandular, sarcomatoid, yolk sac-like, and mixed histological patterns [15, 37]. A rare histologic subgroup of SWI/SNF-deficient neoplasms and the subject of our investigation is SMARCB1-deficient sinonasal adenocarcinoma defined by glandular features. In our limited series, we found that a majority of SMARCB1-deficient sinonasal adenocarcinomas exhibited a dominant eosinophilic pattern with an oncocytoid/rhabdoid appearance, followed by a solid blue-basophilic cell pattern. Glandular structures including tubular, microglandular, cribriform, solid, and signet-ring patterns were identified in all our cases, comparable to other published series also showing intracytoplasmic and intraluminal mucin confirmed by mucin staining [15]. Of interest is the finding of yolk sac tumor-like differentiation with Schiller-Duval body-like structures in 34% of our cases, with both basophilic and non-pink cell appearances.

Three potential pitfalls can be expected in the diagnosis of SMARCB1-deficient sinonasal adenocarcinoma. First, the common eosinophilic-glandular pattern of this tumor could be misclassified as a high-grade non-ITAC. Loss of immunohistochemical SMARCB1 staining, which was uniformly observed in all our cases, is an essential criterion to avoid this diagnostic error. A second differential diagnostic error could be due to the basophilic/blue glands with endometrioid/yolk sac-like structures that might be interpreted as features of ITAC. Although this misinterpretation is more likely due to the focal CK20 and CDX2 positivity seen in these cases, there was a lack of diffuse CK20/CDX2 staining, which, along with the loss of SMARCB1 staining and focal positivity for glypican-3 and/or SALL4, helped in differentiating these entities. Shah et al. also highlighted other differential diagnoses in this setting, such as extragonadal or metastatic yolk sac tumor and metastatic hepatocellular carcinoma [15]. These diagnoses could be inferred from positive staining for glypican-3 and SALL4; however, these entities are extremely rare in the sinonasal tract. Moreover, negative SMARCB1 and other germ cell markers would rule out these diagnoses. Third, the microglandular/cribriform and eosinophilic appearance of cells with focal p63 staining can be interpreted as myoepithelial differentiation. Our cases were entirely negative for S100 and SOX10, which, in combination with SMARCB1, negativity excludes myoepithelial carcinoma.

SWI/SNF-deficient malignancies pursue a highly aggressive clinical course, resulting in widespread disease dissemination either at the time of diagnosis or soon afterwards and causing the death of the patient soon after diagnosis, despite an apparently curative therapeutic intent. Systemic chemotherapy has shown no success so far [23]. To date, satisfactory systemic chemotherapy has not been established for these patients. This disappointing finding underlines the urgent need for effective systemic therapy to allow sufficient intermediate to long-term disease control. However, there are a number of scientific investigations aimed at deciphering the vulnerable molecular sites secondary to SWI/SNF mutation in these tumors [38]. Gene sequencing and molecular subgrouping of each SWI/SNF-mutated tumor will help identify a target suitable for tailored therapy [35]. Recently, SWI/SNF-deficiency has increasingly emerged as pivotal in cancer immunogenicity and hence a promising biomarker when predicting response to immune-checkpoint inhibition therapy utilizing several recently established drugs [23]. Immunotherapy targeting PDL1 [35, 39], employing the PRC2 (EZH2 subunit) inhibitor tazemetostat [15], as well as inhibitors acting against protein kinases, MYC, MDM2/4, and the proteasome are major examples of these efforts [32, 35].

## Summary

In this limited series, it appears that SMARCB1-deficient SNACs show a predilection for male patients, and in contrast to non-glandular tumors, may occur with greater frequency in the nasal cavity. The original diagnosis in most cases of SMARCB1-deficient SNAC was HG non-ITAC and less frequently HG myoepithelial carcinoma or HG ITAC. Differential diagnosis is challenging, but the availability of immunohistochemical antibodies against SWI/SNF proteins represents an emerging effective tool for screening these neoplasms. For the first time, we have shown an NGS-detectable mutation in the *SMARCB1* gene in a subset of cases. Recent advances in molecular profiling have led to major updates and revisions in the classification of high-grade sinonasal carcinomas. Although the majority of these tumors are characterized by aggressive biologic behavior, the identification of these mutations could potentially lead to improved targeted therapeutic options and improved overall disease-specific survival.

## Data Availability

Data supporting the findings of this study are available within the article. The complete datasets generated during and/or analyzed during the current study are available from the corresponding author upon reasonable request.
